# Perspectives on preparing for long-acting injectable treatment for HIV among consumer, clinical and nonclinical stakeholders: A qualitative study exploring the anticipated challenges and opportunities for implementation in Los Angeles County

**DOI:** 10.1371/journal.pone.0262926

**Published:** 2022-02-03

**Authors:** Oluwadamilola Jolayemi, Laura M. Bogart, Erik D. Storholm, David Goodman-Meza, Elena Rosenberg-Carlson, Rebecca Cohen, Uyen Kao, Steve Shoptaw, Raphael J. Landovitz

**Affiliations:** 1 Department of Family Medicine, University of California, Los Angeles, Los Angeles, CA, United States of America; 2 RAND Corporation, Santa Monica, CA, United States of America; 3 School of Public Health, San Diego State University, San Diego, CA, United States of America; 4 Division of Infectious Diseases, David Geffen School of Medicine at UCLA, Los Angeles, CA, United States of America; 5 Division of HIV and STD Programs, County of Los Angeles Department of Public Health, Los Angeles, CA, United States of America; 6 UCLA Center for Clinical AIDS Research and Education, University of California, Los Angeles, CA, United States of America; Fundacion Huesped, ARGENTINA

## Abstract

Long-acting injectable (LAI) antiretroviral therapy (ART) is a novel HIV treatment option for people with HIV. The first LAI ART regimen for HIV treatment received regulatory approval in the United States in January 2021. In February 2020, we collected qualitative data from 18 consumers and 23 clinical and non-clinical stakeholders to catalog anticipated individual-consumer, healthcare system, and structural levels barriers and facilitators to LAI ART implementation in Los Angeles County, California. Thematic analysis was guided by the CFIR implementation science model. CFIR constructs of intervention characteristics, individual characteristics, outer and inner setting, intervention characteristics, and implementation process emerged in analysis. Under intervention characteristics, anticipated facilitators included the relative advantage of LAI ART over pills for adherence and reduced treatment management burden and related anxiety; anticipated barriers included non-adherence to injection appointments, concerns of developing HIV resistance, discomfort with injection and cost. Anticipated facilitators based on individual characteristics included overall acceptability based on knowledge and positive beliefs about LAI ART. Participant noted several characteristics of the outer setting that could negatively impact implementation, such as medical mistrust, external policies, and LAI ART eligibility (i.e., to be virally suppressed prior to initiation). Participants were optimistic about the potential to decrease stigma but expressed that provider willingness for adoption could be hindered by challenges in organizational inner setting related to payment authorizations, increased staffing needs, medication procurement and storage, and provider and healthcare system readiness. Results from this pre-implementation study may inform rollout and scale-up of LAI ART in Los Angeles County.

## Introduction

In the United States (U.S.), approximately 1.2 million people were living with HIV as of 2018 [1]. As of 2019, in Los Angeles County (LA County), the setting of the present study, reported 52,004 people were living with HIV [1–3]. Daily oral antiretroviral therapy (ART) has dramatically decreased morbidity and mortality in people with HIV (PWH) [4]. These benefits are attributable to viral suppression afforded by daily oral ART when used as prescribed; however, at the population level, the rate of viral suppression in LA County was estimated to be 60% in 2019 –well below the UNAIDS target of 95% [2, 5].

Several factors have been shown to limit the ability of people with HIV to achieve and maintain viral suppression when using daily oral ART. Some of these factors include transportation, internalized stigma, medical mistrust as a response to systemic racism, and mental health and substance use disorders [2, 6–12]. Despite significant HIV-related therapeutic and preventive innovation and clinical trial successes, inequities in observed real-world benefits across populations indicate the ongoing need for novel interventions that can address barriers to care.

Long Acting Injectable (LAI) ART is a novel HIV treatment option with the potential to increase rates of HIV viral suppression [13, 14]. The first LAI ART regimen was granted regulatory approval in the U.S. in January 2021 and is commercially available [15, 16]. This LAI ART regimen is comprised of two antiretroviral medications with long half-lives when injected intramuscularly: cabotegravir (CAB), an integrase strand-transfer inhibitor, and rilpivirine (RPV), a non-nucleoside reverse transcriptase inhibitor [13, 14]. Clinical trials demonstrated that CAB+RPV successfully maintains HIV viral suppression in PWH. Current data support the use of the long-acting injectable regimen only for people who have achieved viral suppression from daily oral ART as evidenced by an HIV-1 RNA < 50 copies/ml [13, 17, 18]. Clinical guidelines indicate that LAI for HIV treatment is not recommended for individuals with prior resistance to INSTIs or NNRTIs (the K103N mutation being a notable exception), previous virologic/treatment failure, active Hepatitis B infection, children, adolescents, and women who are pregnant, may become pregnant or breastfeeding [19, 20]. General recommendations require a one-month oral lead in to determine safety and tolerability prior to initiation of LAI ART [21]. The European commission recently approved LAI ART for consumers to use without the requirement of an oral lead in, but such approvals are nonexistent in the United States [22].

Previous studies highlight potential advantages and barriers to LAI ART adoption. The potential advantages of LAI ART for consumers may include convenience, increased confidentiality, and removal of the need for a daily routinized behavior (tablet-taking), as well as the avoidance of drug-drug interactions modulated by first-pass hepatic metabolism. Barriers for both providers and consumers include concerns around safety and efficacy, increased clinic visits (LAI ART obligates monthly injections, compared to recommendations for stable ART follow-up being semi-annually), burden to workflow and cost, and low perceived trustworthiness of the health care system and consequent mistrust of new treatments [23–29]. In particular, Black communities in the U.S., which are disproportionately impacted by HIV, have expressed high rates of mistrust of new medical interventions due to the impact of historical, structural and systemic racism and discrimination in healthcare delivery and medical research studies [30–37]. Moreover, the requirement to achieve HIV viral suppression prior to eligibility for LAI ART may limit the potential benefit to those who are the most challenged by oral ART. Populations that might be expected to most benefit from less frequent dosing, such as people experiencing homelessness, substance use disorders, and mental health disorders, may not be eligible for LAI ART if they are unable to first achieve virologic suppression, even if transiently.

Furthermore, at the organizational level, the provision of LAI ART will require a paradigmatic shift in HIV care delivery for service providers. The current recommended interval for follow-up for stable PWH is 6 months, and monthly dosing (although upcoming secondary approvals are expected to extend the interval to every 2 months) [38]. In this way, the implementation of LAI ART has the potential to increase the number of annual healthcare system contacts required. The level of administrative and/or insurance authorization approvals that will be required may impose additional operational challenges.

Although studies have documented a variety of facilitators and barriers to consider for LAI ART implementation, most prior research has focused primarily on patient-related barriers. The present study uniquely addressed organizational and provider considerations. LA County, the setting of the present research, has many HIV service providers spread across a vast geographic area serving a racially and ethnically diverse population of PWH. LA County is likely to be a microcosm of implementation challenges that can be anticipated across various jurisdictions and geographies in the U.S. Supported by the NIH Ending the Epidemic grant, this manuscript reports findings from a one-year planning project focused on the pre-implementation phase of LAI for HIV treatment. The study assessed the perceived policy, systems, financial, operational, clinical, and consumer-level barriers to and facilitators of rollout and scale-up of LAI ART, from the perspective of clinical and non-clinical HIV providers, healthcare administrators, and other key stakeholders, as well as potential consumers.

## Methods

### Conceptual framework

The Consolidated Framework for Implementation Research (CFIR) model was adopted to guide the study design, assessment, and analysis [39, 40]. The CFIR was used to identify the multilevel (organizational- and consumer-level) aspects that need to be addressed for LAI ART for HIV treatment implementation [41]. The CFIR’s five main domains and multiple constructs used were intended to capture barriers to and facilitators of implementation. The domains we explored included intervention characteristics (e.g. relative advantage of LAI ART over oral ART, perceived adaptability of LAI ART to meet the current local needs of consumers, potential complexity in the steps required to implement LAI ART, and costs of implementation), outer setting (e.g. patient needs and extent to which these needs are prioritized, and influence of external policies such as standardized guidelines and eligibility criteria on LAI ART implementation), inner setting (e.g. structural characteristics and capacity of the organization, the organizational implementation climate, capacity for change, readiness for implementation aided by availability of resources and access to knowledge, and overall perceived relative priority of LAI ART in the HIV treatment continuum), individual characteristics (e.g., knowledge and beliefs about the intervention and personal attributes such as motivation to use LAI), and also the implementation process (characterized by plans to ease implementation and engagement of key stakeholders with critical roles in the implementation process). The CFIR has been previously used to study the implementation of new technologies and clinic-based interventions for PWH and to explore the system-level challenges and facilitators to the implementation of these interventions [29, 42, 43]. See Table 1 for identified themes and representative quotes within each CFIR domain.

**Table 1:** CFIR constructs and representative quotes.

CFIR construct	Subtheme	Consumer stakeholder quote	Clinical/non-clinical stakeholder quote
**Intervention characteristics**			
**Relative advantage**	Adherence	“I think that that would be so awesome when that gets approved because for me, I would prefer to do a long acting injectable instead of having to take a pill every day. . .Being adherent has always been a struggle for me. When I was younger just because of a lot of different reasons. But now in my older age, I am busy, I work a lot. I’m always on the go and sometimes I forget. So I won’t have to worry about it.” (Consumer stakeholder)	“I think an injection monthly, maybe every two months, however they get extended to, would be incredible for folks who battle with adherence.” (Clinical stakeholder)
**Relative advantage**	Treatment management	“As of right now I’m in a regimen of one pill a day. . .So I think if it will be every month, it will save a lot of time, a lot of worries, a lot of preoccupation.” (Consumer stakeholder)	“I think it’s going to be great, but I think, as someone else stated, as a choice for people, particularly people who have pill fatigue, and people who have difficulty with taking their medication” (Non-clinical stakeholder)
**Perceived adaptability and complexity**	Resistance	“I travel. I might be in different states, different countries in a month. What if something happens and I’m stuck? And I can’t get to where I need to be to get my injections? So then that’s just like not taking your medicine. So what kind of side effects or resistance am I going to have when I get back? It’s like, oh man, you’re resistant now and I can’t afford to be resistant to anything because I’ll die." (Consumer stakeholder)	“I think all these concerns bring up this underlying specter resistance. And so if we’re missing doses, are we going to start having a lot of resistance.” (Clinical stakeholder)
**Perceived complexity and adaptability**	Treatment frequency	“I do see some drawbacks with having to schedule an appointment, go to the appointment, for those that are busy, or those that have other challenges in their life.” (Consumer stakeholder)	“Because I think that’s going to be the big problem. . .you’ve been complaining when you have to come in every four months. Now you’ve got to come in every month. I think this, if we have it every three months, it’d be goldmine. But at once a month, it’s going to still be hard for the right patient.” (Clinical stakeholder)
**Perceived complexity and adaptability**	Treatment control	“. . .And I know what happens in a national disaster! Everything closes. Your doctors closes, ain’t no ambulance, ain’t no hospitals, ain’t nothing. You’re on your own for some time. It could be months. It could be several months before we get any type of help. But guess what? I got enough supplies to last me until something comes up, the government or whatever aid comes to us, you know what I’m saying? I got my own back. With this right here, who got my back? Do you really think the doctor’s going to be available to me to give an injection during an earthquake or other national disaster that’s going to happen?” (Consumer stakeholder)	“You relinquish, as a person with HIV you relinquish that control that you would [have] taking your pills. . .Now you have to go to a place where you have somebody [administer] the medication. . .” (Non-clinical stakeholder)
**Key features**	Pain/Side Effects/Comfort with Shots	“I don’t like doing shots. I hate to get them when I go to the doctor. And then you tell me I have two of them? One will be enough for me. You talking about two? I’ll stick with my pills.” (Consumer stakeholder)	“And I think because of the long half-life, that might be a concern because with a pill if you feel like you’re having side effects, you can just stop. But with the injectable, I mean you’re kind of stuck for at least a month, right? So I could see people being hesitant for that reason.” (Non-clinical stakeholder)
**Cost**	Financial concerns (e.g., cost, billing, insurance, pre-authorizations)	“I think it’s a deal breaker if I can’t afford it. I mean, definitely I’m not going to do it, I mean I can’t do it, if it’s not really in my budget, or my insurance doesn’t cover it, or whatever. . .I mean, that’s probably a major consideration.” (Consumer stakeholder)	“I think the prior authorizations. . .is where we’re going to get crushed on this. And I would assume that, compared to the oral medicines, I would assume it’s going to be a more expensive drug. . .And if it is true, that means a lot of work on our end to do prior authorizations. Is it just a one-time prior authorization, or are there going to be. . .Is it going to be every month, every three months? When people change insurances, you’re going to have to do it again? That’s a concern.” (Clinical stakeholder)
**Individual characteristics**			
**Knowledge and beliefs**	Organizational Support/Willingness	N/A^a^	“Well, I think it’s a positive contributor to our materials to treat our patients. It’s not going to be with every patient, but there will patients that it will be their ideal treatment. (Non-clinical stakeholder)
**Self-efficacy**	Knowledge of LAI	N/A^a^	“And also just having a little more information about it like we were talking about like window periods of how long people could go without it. I don’t think there’s enough research yet or enough information about long-term effects and all those things about how people will interact with them. And how they could potentially affect their lives. Just having all that on hand of course is helpful.” (Non-clinical stakeholder)
**Outer setting**			
**Patient needs and resources**	Addressing Medical Mistrust	“That is actually a huge thing in the Black community. Mistrust or a lack of trust in the healthcare field. . .So that could definitely be an issue that could get in the way…” (Consumer stakeholder)	“I think most persons would want to see somebody else looked at it before. . .because remember AZT, because when I hear some of the stories that they told me, they saw their friend dropping and dying and stuff like that. I think a lot of them would be on the sideline trying to see okay, let me see who is going to drop first. It’s kind of like see if it works.” (Non-clinical stakeholder)
**Patient needs and resources**	Addressing Stigma	“…it was in the bathroom and they, my friend, went in there and saw it and he was like. . .next thing I knew the phone was ringing off the hook. Now he went and told everybody because he knew what it was. And I was like, "Well, what are you doing going through my medicine cabinet in the first place?" … So I think that it would be more advantageous to go on ahead, get the shot once a month and be done with it. And then that’s your business. It’s nobody else’s business.” (Consumer stakeholder)	“a lot of patients we find just the daily routine of taking a pill every day is a reminder that they are ill, and I think the injectable has an option to take that stigma away, or at least for that personal, internal stigma away from those people living with HIV, who kind of feel that burden” (Clinical stakeholder)
**Patient needs and resources**	Structural barriers	“I think that would save a lot of people’s lives. In the long run, this is not for everybody, but I’m saying that the people that really need it are those type of people. And poor people who sell their medicine because they don’t have food, water, or whatever. And the people who are addicts, they can’t sustain a natural well-being of taking medicine every day.” (Consumer stakeholder)	“I think if we are going to target the homeless population, one barrier that I foresee is transportation, and a lot of times they’re losing their phones, they get their stuff stolen. Getting a hold of them and making sure they continue to come to their appointments is very challenging. I foresee that as a big barrier.” (Non-clinical stakeholder)
**External policies**	Eligibility Criteria	“…the requirement to be unattainable to start, I get it. But it’s just not a reality friendly.” (Consumer stakeholder)	“It’s just going to benefit people who are already doing really well. And when we think about where we need to make advances, that’s not the group, right? It’s the folks that aren’t virally suppressed. It’s the folks that are going to have a hard time making it to the doctor’s office on a monthly basis.” (Non-clinical stakeholder)
**External policies**	Clear guidelines	N/A^a^	“And I always think about, how do we offer any new technology, or new treatment options, in medicine? And I think the Community Advisory Board is a good thing, clinic or institutional guidance, or guidelines. Ultimately the DHHS guidelines is, ‘Is this a preferred therapy or not?’ And I think that’s a powerful tool to both disseminate the information amongst providers, so we’re all using standard quality therapies, and maintaining quality in our prescribing.” (Clinical stakeholder)
**Inner setting**			
**Implementation climate**	Organizational Acceptability	N/A^a^	“But when it’s just about supporting choice and acknowledging that people have fatigue that feels really good. But I think for clinic administrators who, they’re like, they’re getting the same thing. I think it would be a really hard argument with additional cost. And new systems and burdens and asking nurses to do lots of shot teaching potentially and shot administration. I love our clinic administrators but I don’t know if they’d go for it.” (Non-clinical stakeholder)
**Relative priority**			
**Readiness for implementation (available resources and access to knowledge)**	Staff preparedness	N/A^a^	“I want to address something that we haven’t addressed and I think it’s about the training for the healthcare worker force. I think implementing the program for injectables of HIV clinics has to be very well thought in regard to training and capacity building. Not only from the clinic administrators but also from the persons dispensing and applying the medications.” (Non-clinical stakeholder)
**Structural characteristics**	Staff capacity	N/A^a^	“It’s also changing the flow of clinics. Most of our folks, I don’t know, at least in our clinics, we’re not seeing people monthly unless it’s the beginning of their diagnosis. You’re talking about an influx actually of new visits potentially. Whether our clinics can handle that, whether we have the staffing. . .” (Non-clinical stakeholder)
**Structural characteristics**	Physical Infrastructure and Supply Management	N/A^a^	“. . .there’s going to be some logistical issues, too. . .I mean, how are we getting it? Is it coming from the pharmacy? Do they get it, bring it to the clinic, you inject them? Or does it come straight to the clinic? So your clinic now has to set up to store this. Do we order it ourselves? I mean. . .Many of us don’t have our own pharmacies…” (Clinical stakeholder)
**Implementation climate (learning climate)**	Provider bias	N/A^a^	“. . . I would hope that we could also anticipate maybe some biases in terms of providers about to give the injectable. Then I would wonder okay, if we’re worried about adherence and things like that, is that going to skew providers to being like, ‘oh, this client has really bad history with adherence. I’m not going to give it to them,’ even though maybe the injectable is exactly what is needed to deal with that issue.” (Non-clinical stakeholder)
**Process**			
**Planning and engaging**	Marketing recommendations	“I think it’s nice to have two or three things to position equally the immediate benefits. And you share what is good immediately and you know that, you’re actually not reading many many things, maybe three things…” (Consumer stakeholder)	“. . .the peer to peer strategy. I think [it] is incredibly effective, particularly when using people within a community who are essentially popular, the popular kids. You tell them, ‘Hey, we’ve got this. Could you talk to your friends and stuff about it?’ I think when they’re the ones to push the message, especially for people of color, then it’s a little more digestible.” (Non-clinical stakeholder)
**Planning and engaging**	Alternative Staff to Deliver LAI ART Treatment.	“I envision the larger specialty AIDS clinics setting up a streamlined process where they know that people are going to have to do this every month, and that there’s a special thing. Because my guess is that a nurse can just give it. And then they would just assembly-line it almost.” (Consumer stakeholder)	“There’s a much wider group of people who can, I assume can administer, so you have nurses and pharmacists and pharmacy techs even…” (Non-clinical stakeholder)
**Planning and engaging**	Treatment education and Adherence support	“Doctors need to communicate with us on everything whether they’re an HIV doctor or not. Primary doctors. It’s part of their responsibility.” (Consumer stakeholder)	“I think one of the easy thing to do is have an app or a program where persons can also go and check in to have kind of [a] support system where they can possibly talk to each other, just to see how somebody else is doing. If they’re having any sides, something that they’re uncomfortable with that they can share it in that space. That would be a great tool to have. Or maybe we can do the regular old stuff where we have once in a while, like a monthly meeting, where people check in like a support group kind of to help them through the process as well.” (Non-clinical stakeholder)
**Planning and engaging**	Pilot studies	N/A^a^	“But there can be, I think, best practices and guidelines that we should develop, I think, prior to that. . .This trial, are they looking at real world applications? What are we going to draw from? Not just rolling this out, for patients, just for, in a sense, depending on their desire for adherence, and sticking to the program, too. So we have to study that, too.” (Clinical stakeholder)
**Planning and engaging**	Research trials	N/A^a^	“And also just having a little more information about it. . .like window periods of how long people could go without it. I don’t think there’s enough research yet or enough information about long-term effects and all those things about how people will interact with them. And how they could potentially affect their lives.” (Non-clinical stakeholder)
**Planning and engaging**	Innovative Ideas	“Also, this is not the first time injectable treatment of disease has been introduced to the market. I’m sure diabetes went through the same cycle. At first there were pills or whatever, and then Metformin, and God knows what else. And doctor whatever performed injection. Then it was self. . . So, they went through a cycle and I’m sure they did a marketing campaign or whatever, however that was introduced to the public, we can replicate that process. Because that was successful. Everybody knew about it.” (Consumer stakeholder)	“I think there’s a lot of innovative options that we’re seeing in all different therapies, and it’s going to come down to the different patient groups, and their interests. I think the whole home delivery is one thing. I think pairing it with a support group once a month is another thing. A group clinic. Pairing it with food bank is another. So finding the needs, whether they’re psychosocial, or financial, or just mobility, and pairing the needs with the medication delivery.” (Clinical stakeholder)

^a^N/A is indicated where a specific construct did not apply to consumer stakeholders or consumer stakeholders did not provide discussion content.

### Focus groups

Consumers, clinical stakeholders, and non-clinical stakeholders were invited to participate in focus groups in LA County, California. Consumer participants were recruited using flyers distributed to agencies that provide HIV-related social and clinical services. Consumer participants were HIV-positive individuals aged 18 years or older who were enrolled in the LA County Division of HIV and STD Programs Medical Care Coordination program. Separately, current clinical and non-clinical stakeholder partners were identified and invited to participate by email. Stakeholder recruitment focused on leaders and key informants from healthcare clinics, community-based organizations, and government organizations, such as HIV care and service providers, administrators, policymakers, and funders. Interested individuals were offered participation.

Two focus groups with a total of 18 consumers, two focus groups with a total of 23 clinical and non-clinical stakeholders, and one semi-structured interview with a non-clinical stakeholder were conducted in February 2020. All activities were completed in-person at three locations in LA County. The interview session was initially scheduled as a focus group but was adapted to a semi-structured interview because only one participant attended. One of the stakeholder focus groups was with a combination of clinical and non-clinical stakeholders, while the other included clinical stakeholders only.

Prior to the beginning of each session, participants received an information sheet about the study and provided verbal consent to participate and be audio-recorded. They completed a brief questionnaire that collected socio-demographic data and information regarding prior familiarity and experience with LAI ART. To ensure that all participants had basic background information, a study clinician also provided a brief overview of LAI ART before the session began, including information on its efficacy and possible side effects from clinical trial data.

The sessions ranged from 50–120 minutes in length and were conducted by a trained facilitator using a semi-structured focus group guide (S1 Table). Consumer participants were each compensated $50 cash for their participation. The sessions were digitally audio-recorded, transcribed, and stored on a secure server for analysis. See characteristics of focus group participants in Table 2.

**Table 2:** Characteristics of focus group participants.

**Consumer stakeholders**	
**Sociodemographic characteristics**	**Mean or n (%)**
Age (SD, M, Range)	[11.53, 52.2 years, 20–69 years]
Education	
Less than high school diploma	1 (6)
High School Diploma or GED	3 (17)
Some college, but no degree	7 (39)
College degree	4 (22)
Graduate degree	3 (17)
Sex assigned at birth	
Female	5 (28)
Male	13 (72)
Gender identity	
Female	5 (28)
Male	13 (72)
Sexual orientation	
Bisexual	3 (17)
Gay/Lesbian (homosexual)	11 (61)
Straight (heterosexual)	4 (22)
Race/Ethnicity	
Black (Non-Hispanic/Latinx)	7 (39)
Hispanic/Latinx	9 (50)
White (Non-Hispanic/Latinx)	2 (11)
**Knowledge and perception of LAI**	**n (%)**
Heard of LAI	
Yes	11 (61)
No	7 (39)
Know anyone who has used LAI	
Yes	1 (6)
Don’t Know/Not Sure	1 (6)
No	16 (89)
Likelihood of using LAI	
Don’t Know/Not Sure	4 (22)
Very Likely	8 (44)
Likely	4 (22)
Not At All Likely	2 (11)
**Clinical and Non-clinical Stakeholders**	
**Sociodemographic characteristics**	**Mean or n (%)**
Age [SD, M, Range]	[12.47, 45.65 years, 28–75 years]
Education, Highest Degree n (%)	
High School diploma	1 (4)
Associate degree	1 (4)
Bachelor’s degree	6 (26)
Master’s degree	2 (9)
Doctoral degree	13 (57)
Roles	
Clinical provider	14 (61)
Non-clinical provider	8 (35)
Unreported	1 (4)
Time in Role (Range)	[1.5–25 years]
Sex Assigned at Birth	
Female	6 (26)
Male	17 (74)
Gender Identity	
Female	6 (26)
Male	17 (74)
Race/Ethnicity	
Asian (Non-Hispanic/Latinx)	4 (17)
Black (Non-Hispanic/Latinx)	8 (35)
Hispanic/Latinx	6 (26)
White (Non-Hispanic/Latinx)	5 (22)
**Knowledge of LAI**	**n (%)**
Heard of LAI n (%)	
Yes	23 (100)
No	0 (0)

### Data analysis

A study team member compiled the questionnaire responses and calculated descriptive statistics. Recordings from the five sessions were transcribed by a certified transcription service and checked for quality assurance. Two study investigators (LMB and EDS) performed inductive thematic analysis, independently reading the transcripts multiple times and looking for patterns to identify general themes and generate initial codes in accordance with standard qualitative content analytic methods [44–46]. Guided by the main CFIR components, LMB developed a preliminary results summary and an initial codebook from the transcripts, which EDS then expanded to list each theme and subtheme with detailed descriptions, inclusion and exclusion criteria, and typical examples. The codebook was further refined through an inductive and reiterative process with the two investigators and two coders (OJ and ERC) that included reliability scoring and discussion of inconsistencies until consensus was reached. Twenty percent of the transcripts were coded and tested for reliability and consensus.

Once acceptable inter-coder reliability was reached (Cohen’s Kappa = 0.89), the codebook was finalized, and the two coders used Dedoose Version 8.3.17 qualitative data management software to facilitate the coding of the five transcripts. After all data were coded, the two coders and one investigator (EDS) reviewed the coded passages to identify key themes regarding overall LAI acceptability, barriers to and facilitators of LAI ART, and messaging and implementation recommendations for LAI ART rollout, overall and by participant group. The main themes in this study were identified and aligned to possible CFIR model’s major domains; intervention characteristics, individual characteristics, inner setting, outer setting and the process-implementation strategies and recommendations.

### Ethics statement

The study was approved by the University of California Los Angeles Institutional Review Board. UCLA IRB #19–001381. Verbal Informed consent was obtained from all individual participants included in the study. Participants were provided an information sheet about the study, and were given the opportunity to ask questions. Participants gave their verbal consent in place of written consent to participate. The informed consent procedure was approved by the UCLA IRB.

## Results

### CFIR constructs and qualitative themes

The main CFIR constructs were used as a basis to organize the analysis and to identify emergent themes. The CFIR constructs and representative quotes for each construct are shown in Table 1. Participants indicated that the adoption and successful implementation of LAI ART for HIV treatment is influenced by factors within the intervention characteristics, individual characteristics, outer setting, inner setting, and implementation process.

#### Intervention characteristics

*Relative advantage*. Participants generally perceived LAI ART as a relative advantage over oral ART. Participants expressed that LAI ART may be easier for some consumers to adhere to than daily oral ART, especially for consumers who experience adherence challenges e.g., forget to take their pills, experience treatment fatigue due to the high pill burden. Across groups, there was a shared perception that LAI ART may contribute to reduced treatment management burden for consumers, decreased treatment frequency, and decreased responsibility for treatment management.

*Perceived adaptability and complexity*. Perceived adaptability of LAI ART to meet local treatment needs and the potential complexity required to adopt LAI ART emerged as key constructs. Adaptability to treatment requirements, especially around the monthly clinic visits required for LAI ART, was a key perceived barrier to willingness to adopt LAI ART among all groups. Consumer, clinical, and non-clinical stakeholder participants recognized the potential benefits of LAI ART for vulnerable populations but also expressed concerns about non-adherence to LAI ART appointments and the potential drug resistance that may result from prolonged dosing delays, especially for populations who experience mental health, substance use issues, or structural barriers, such as inadequate transportation access, homelessness, and poverty. The perceived likelihood of LAI ART to disrupt workflow and place an increased demand on staff capacity within a clinic was expressed by clinical and non-clinical stakeholder participants.

*Key features*. Key features of LAI ART, specifically the needle-based injections (and related fear of injection site pain) as well as potential side effects—were expressed as barriers to LAI ART use across groups. Participants commented that some consumers may fear the anticipated pain of two large-volume injections in the buttocks. Also expressed was personal concern about potential short-term and long-term side effects that may be discovered post-implementation of LAI ART, which was not previously identified by limited clinical trial data. Clinical and non-clinical stakeholder participants noted that the long half-life of an LAI ART dose may make the prospect of experiencing side effects more worrisome for consumers. They believed that some consumers may need to see how early adopters react to LAI ART before feeling comfortable trying it themselves.

*Cost*. The cost of implementation emerged as a barrier that had the potential to influence LAI ART implementation. Across groups, participants expressed concern about financial barriers to LAI ART use. Consumer participants generally assumed their insurance would cover the cost but noted that cost would be a barrier for many consumers if LAI ART is not adopted by formularies. Clinical stakeholder participants expressed concern around the cost of the drug and the related insurance preauthorization required.

#### Characteristics of individuals

Participants’ narratives suggested that the adoption and successful implementation of LAI ART for HIV treatment will be influenced by behaviors, norms, and beliefs of individuals within and organization.

*Knowledge and beliefs*. Clinical and non-clinical stakeholders’ knowledge and beliefs around the intervention conveyed support for and willingness to adopt LAI ART as a new treatment option. Participants expressed that many providers would appreciate having another HIV treatment tool available.

*Self-efficacy*. Clinical and non-clinical stakeholders, however, expressed concern around capabilities and self-efficacy-which is dependent on the ability of the provider to perform specific actions within a specific context [47]. Participants noted that the lack of clarity around the efficacy, safety, and optimal context of use for LAI ART including knowledge of the treatment half-life and long-term effects, could serve as a barrier to adoption of LAI ART.

#### Outer setting

**Outer setting** are the external influences the healthcare system has on an intervention, especially in the ability of the organization to prioritize patient needs, and ensure external policies and procedures are in place to aid successful implementation. Medical mistrust in the healthcare system as a response to systemic racism and discrimination was perceived as a possible outer setting barrier to LAI ART use, particularly for Black consumers. Consumer and non-clinical stakeholder participants expressed that consumers who distrust the medical system may be wary of trying a new treatment, especially one that requires reliance on healthcare providers.

*Patient needs and resources*. Patient needs for addressing stigma emerged as a key facilitator of LAI ART use across groups, as participants noted that LAI ART use may help ease the burden of HIV-related internalized and social stigma. Consumers felt that LAI ART would help to reduce the shame and constant reminders of HIV illness associated with taking daily oral ART, and the fear of pills potentially being discovered by other people. Clinical and non-clinical stakeholder participants also noted by a participant that increased clinic visits may influence the likelihood of perceived stigma for consumers who do not want to be seen at HIV-associated locations. Patient needs around addressing structural barriers emerged as a potential barrier. Participants noted that structural barriers such as the lack of access to transportation and housing may hinder consumers from successfully keeping the required LAI ART monthly appointments. **External Policies.** The lack of external policies was also highlighted as potential barrier to LAI ART implementation. Participants across groups expressed concern that consumers who could potentially benefit most from LAI ART, such as those who are non-adherent to oral ART, might not initially be eligible for LAI ART based on the current eligibility criteria of viral suppression prior. Clinical and non-clinical stakeholder participants further expressed that the eligibility requirement appears to exclude newly diagnosed, treatment-naive consumers for whom LAI ART may be the preferred option. Participants questioned the representation of certain populations in the research that determined LAI ART efficacy and eligibility, including consumers of color and the required exclusion of pregnant and breastfeeding women. Clinical stakeholders indicated a need for external clear guidelines and best practices for LAI ART use, to ensure standardization around identifying appropriate consumers for LAI ART and prescribing the treatment.

#### Inner setting

*Implementation climate*. Within the inner setting of the organization, the implementation climate, i.e., how receptive the organization is to adopting LAI ART for HIV treatment, was a key consideration. Some clinical and non-clinical stakeholder participants commented that organization administrators and providers may be wary of the increased complexity of adopting LAI-ART use, particularly for consumers who have been successful with pill-based ART.

*Readiness for implementation*. Organizational readiness for implementation was highlighted as a potential barrier to LAI ART implementation. Clinical and non-clinical stakeholder participants discussed concerns around the need for establishing effective and streamlined procedures for prior authorizations and billing. Clinical stakeholders also commented that payment procedures must account for regular changes in consumer insurance coverage and missed LAI ART doses. Clinical and non-clinical stakeholder participants expressed concern about provider and staff preparedness, specifically around staff receiving enough training, education, and information to be prepared for LAI ART implementation. Participants further conveyed the need for staff to understand the science behind LAI ART prior to implementation, and for organizations to establish and follow clear operational protocols to minimize confusion and errors early in implementation. The learning climate within the organization was a significant consideration, as some non-clinical stakeholder participants noted concerns about provider bias especially in objectively determining the treatment needs of consumers for whom they choose to adopt LAI ART.

*Structural characteristics*. Structural characteristics such as staff capacity and need for physical space emerged as key organizational barriers to implementation. Clinical and non-clinical stakeholder participants felt concerned about the staffing needed for LAI ART implementation, including responding to increased consumer volume, procuring, and monitoring supply, providing treatment education and administration, and managing missed appointments. Clinical and non-clinical stakeholder participants commented on the need for physical space to accommodate increased consumer volume and store LAI ART supply, storage equipment and protocols. Participants expressed particular concern for clinics without on-site pharmacies regarding their capacity to manage LAI ART supply. Furthermore, compatibility in the anticipated changes to clinic workflow were also perceived as an implementation challenge.

#### Process

*Planning and engaging*. In the pre-implementation process, participants suggested involving a series of steps to aid implementation, such as planning, in terms of putting the proper steps in place and engaging key entities in preparation for implementation. Engaging community members to deliver the messages around LAI ART emerged as potential facilitators for LAI ART implementation. Across groups, participants expressed that medical professionals should deliver scientific information, but that consumer voices should be at the center of LAI ART promotional campaigns. Participants emphasized that community engagement would be critical for effective message development and implementation, and for establishing buy-in and reducing mistrust of LAI ART among priority populations.

Suggested promotional platforms included TV commercials, billboards, bus stop advertisements, waiting room flyers, community presentations, and social media advertisements, as well as direct outreach programs to reach priority populations that may not have access to mass communication platforms. Consumer participants highlighted the value of simple, short messages that promote LAI ART benefits, such as “imagine not having to take a pill every day,” “simplify your life,” and “now HIV treatment can be even easier.”

Planning to implement treatment education and adherence support were also noted as critical facilitators of successful LAI ART implementation. Across groups, participants emphasized the need for LAI ART treatment education and adherence supports for consumers. It was mentioned that medical doctors should be engaged to educate consumers. Education should include general information about LAI ART, what to expect regarding the injection and potential side effects, the treatment interval, and other factors that may influence a consumer’s treatment decision. Suggestions included reminders, incentives, and consumer support groups. Clinical stakeholder participants also discussed the need for electronic medical record (EMR) modifications to support consumer tracking and reminders.

Engaging critical stakeholders such as clinic administration and staff in the implementation process of LAI ART could potentially facilitate success in implementation. Clinical and non-clinical stakeholder participants recommended that alternative non-physician staff, including nurses and pharmacists, be allowed to deliver LAI ART treatment. Participants were uncertain about whether medical assistants would be equipped to deliver LAI ART and suggested additional training would be needed. It was noted that clinicians may need to be available on-site regardless of who delivers LAI ART in case of reactions.

Engaging external change agents such as county, research institutions and organizations to prepare for implementation emerged as considerations. Clinical and non-clinical stakeholder participants recommended that research trials and demonstration projects with priority populations be administered in specific settings to inform the development of guidelines and standardized procedures for LAI ART implementation in LA County. Participants highlighted the importance of research to study the flexibility of the dosing interval’s “window period” and assess the long-term safety of LAI ART.

Participants across groups proposed innovative strategies that involve engaging various change agents to facilitate use of LAI ART across the implementation process. Offering LAI ART at delivery locations outside of traditional HIV clinic settings, such as infusion therapy sites or co-located with substance abuse treatment, food banks, pharmacies, and other services for priority populations, was discussed. Home visits by healthcare providers or nurses were also suggested to mitigate consumer barriers to LAI ART use. Additionally, participants recommended exploring the possibility of self-injection, looking to home testosterone injections as models, and noted that this may need to await data from safety and bioequivalence of injection into anatomic sites other than the buttocks. They advised learning from the implementation of injectable therapies for other conditions such as psychotic disorder and substance use treatment as LA County works towards implementation of LAI ART.

## Discussion

This pre-implementation study examined the willingness of consumer, clinical, and non-clinical stakeholders to adopt LAI ART as a treatment option and identified potential barriers to and facilitators of LAI ART implementation in LA County. Consistent with the CFIR implementation science model, we found that intervention characteristics, individual characteristics, outer setting, inner setting, and implementation process all emerged as important pre-implementation considerations [48, 49].

Both consumers and clinical providers expressed willingness to adopt LAI ART as a treatment option. Adherence to LAI therapies, compared to daily oral ART-pills, was highlighted as a potential major relative advantage that could facilitate LAI ART implementation. Patient needs influenced by the outer setting include the need for culturally appropriate and affirming care with competent providers in a welcoming environment to address stigma, and medical mistrust. Barriers centered on concerns around cost, preauthorization, system workflow, staff capacity needs, and organization/provider preparedness. Facilitators to the implementation process included treatment education/support, and the development of LAI ART treatment guidelines/policies. Previous studies have found similar barriers to and facilitators of LAI ART implementation, and this study further highlights implementation barriers on the consumer and provider level that may not be entirely exclusive to LAI ART implementation [23–29].

Adherence, vis a vis an individual’s ability to achieve and maintain virologic suppression, is a critical priority in ending the HIV epidemic [50–52], and an important consideration in the implementation of LAI ART. Introducing resources to address mental health, substance use disorders, and structural factors (e.g., homelessness, poverty, incarceration, and transportation [53–56]), and developing comprehensive consumer education and treatment support programs, such as peer support groups, linkage to care services, case managers, and patient navigators, spearheaded by key leading health agencies and clinic administration, will be advantageous [57–60]. Furthermore, tailored behavioral interventions and treatment support strategies optimized in the context of LAI ART, and rooted in community engagement and partnerships, are likely to be critical in addressing many of the challenges to adherence that participants expressed [61–64].

The potential of LAI ART to address stigma was generally perceived to be a key facilitator for implementation [24, 58]. Conversely, it was conveyed that the high frequency of office visits for LAI ART may increase the likelihood of perceived or experienced social stigma, therefore precautions at the clinic-level are needed, possibly through organized scheduling of clinic appointments, restructured office spaces, and less crowded waiting rooms. Administering LAI ART at alternate locations other than the traditional HIV care settings, such as pharmacies and infusion centers, could also be helpful [65, 66]. Ultimately, consumer-centered discussions about LAI ART will be necessary to ensure consumers are aware of the potential for stigma and are able to choose the best modality for their treatment.

Fostering trust between consumers and their providers is important, especially in Black communities that have faced a history of systemic racism and negative healthcare experiences [32, 34, 36, 67–69]. A positive consumer-provider relationship is correlated with increased satisfaction with care, health-seeking behaviors, and improved adherence to treatment [68, 70]; Thus, provider trainings on humility and psychosocial communication training may be helpful [71], combined with healthcare organizational efforts to be more trustworthy, beginning with authentic community engagement [72].

Providers showed an overall willingness to adopt and implement LAI ART as an option; however, concerns around readiness for implementation and preparedness to provide LAI ART to consumers. The findings underscore the need for adequate provider training and education prior to and in the early phases of implementation [73]. Provider training improves health outcomes for the prevention and treatment of HIV and will be a key step in improving viral suppression rates for people using LAI ART [74, 75]. To achieve success in implementation, health care organizations will need to recognize LAI ART as an acceptable treatment option and establish standard guidelines, recommendations, and educational curriculum around LAI ART, to include pharmacology, consumer eligibility, and prescription practices for treatment delivery staff. Extensive education around this new treatment tool should be made available for providers to feel comfortable discussing the medication with consumers.

Organizational preparedness is key to addressing potential implementation barriers around cost, insurance pre-authorizations, staff capacity needs, and medication procurement and storage. Addressing staff capacity needs because of anticipated changes to clinic workflow will require increased efficiency and protocolization within the clinic systems. Similarly, streams of authorization and payment systems should be established, and out-of-pocket costs minimized for the most vulnerable populations. Additionally, a reimbursement model will need to be developed to support additional staff time and headcounts. Medication procurement and storage processes need to be streamlined and the administrative burden minimized for both clinic staff and consumers.

Currently, early uptake of LAI ART has been challenged by a cumbersome system of product procurement and delivery, confusion around insurance coverage and lack of experience with implementation. Although this study was conducted prior to the COVID-19 pandemic, it will be important to additionally integrate contingency plans for future and unforeseen effects of pandemic-related disruptions on wide-scale rollout, bearing in mind the required treatment frequency for LAI ART for HIV treatment amidst pandemic safety concerns and policies. Furthermore, engaging key players in the implementation process of LAI ART to conduct pilot studies using differentiated care models [76–78], during the early stage rollout of LAI ART will further help evaluate the feasibility of implementation and identify additional logistical needs and facilitators.

This study has limitations that should be noted. The analyzed themes were based on responses from a small sampling of stakeholders, and participants’ responses may have been influenced by the group setting. Although purposively recruited, the study sample did not include individuals from younger, transgender, and sex worker populations which may limit interpretation of study findings. The study was based exclusively in LA County, which may limit the generalizability of the study to other geographic areas.

## Conclusion

This research study suggests that the success of LAI ART implementation will depend largely on extensive preparation at the public and private levels to anticipate and address potential challenges. Stakeholder engagement, partnership building, effective communication, and coordination across agencies including county, private, clinical, and pharmaceutical providers, who are responsible for clinical care, funding, and formulating policies around HIV treatment is vital to supporting successful large-scale LAI ART implementation. Engaging community stakeholders, especially vulnerable populations—prioritizing treatment support, establishing provider guidelines for LAI ART, and maximizing system efficiencies to minimize staff burden are important considerations. Furthermore, the results of ongoing studies that are evaluating LAI therapy in previously non-adherent populations will inform whether LAI therapy will be able to safely and effectively be leveraged in populations most in-need of this mode of ART delivery. Our findings are helpful in charting the data gaps and implementation considerations for HIV service providers, community members, and other stakeholders as they anticipate scale-up of LAI ART in their jurisdictions across the nation.

## Supporting information

S1 Table Qualitative focus group guide on barriers and facilitators to the implementation of long acting injectable for HIV treatment. (DOCX)
