# Supplementary material for: Perspectives on preparing for long-acting injectable treatment for HIV among consumer, clinical and nonclinical stakeholders: A qualitative study exploring the anticipated challenges and opportunities for implementation in Los Angeles County
Source: PLoS One. 2022 Feb 3;17(2):e0262926. doi: 10.1371/journal.pone.0262926 (PMC8812879; doi:10.1371/journal.pone.0262926)
Supplement: S1 Table — (DOCX) [file pone.0262926.s001.docx]

S1 Table. Qualitative focus group guide on barriers and facilitators to the implementation of long acting injectable for HIV treatment

| **Topic** | **Example Focus Group Questions** | |
| --- | --- | --- |
|  | **Clinical and non-clinical stakeholder participants** | **Consumer stakeholder participants** |
| **General perceptions** | What do you think of long-acting injectable ART overall?  How much do you think long-acting injectable ART should be provided to people with HIV? Why or why not? | What do you think of long-acting injectable ART overall?  How much do you think long-acting injectable ART should be provided to people with HIV? Why or why not?  Would you prefer to take long-acting injectable HIV treatment for HIV or stick to daily pills? Why? |
| **System/clinic/provider-related barriers/facilitators** | How easy or difficult do you think it would be to provide long-acting injectable ART at your clinic/organization? Why?  What are some of the specific barriers or challenges that you foresee in rolling out long-acting injectable ART in your clinic/organization? | ^a^N/A |
| **Patient-related barriers/facilitators** | What kinds of patients do you think would be good candidates for long-acting injectable ART?  What types of patient-related barriers do you think there are to using long-acting injectable ART?  How can barrier be addressed?  How much do you think people with HIV in LA County would want to take long-acting injectable ART? Why or why not?  What factors might help or facilitate patients living with HIV engage in long-acting injectable ART?  What types of adherence support would be needed to ensure that patients follow long-acting injectable ART schedules? | What kinds of patients do you think would be good candidates for long-acting injectable HIV treatment?  What do you think would keep people like you from using long-acting injectable treatment for HIV?  How do you think these barriers can be overcome to help more people to take long-acting injectable HIV treatment? How can each barrier be addressed?  What do you see as the differences of using long-acting injectable HIV treatment compared to taking a daily pill? |
| **Promotion of LAI-ART** | What ideas do you have about how providers should introduce and discuss long-acting injectable ART with patients?  If we were to create messaging or a campaign to promote long-acting injectable ART among people living with HIV in Los Angeles County, what types of messaging would you recommend? Who should deliver the messages? What wording would you use to convince people living with HIV to try LAI-ART? | How do you get advice that you trust about HIV treatment? From doctors, case managers, family, friends, the internet?  If we were to create messaging or a campaign to promote long-acting injectable ART among people living with HIV in Los Angeles County, what types of messaging would you recommend? Who should deliver the messages? What wording would you use to convince people living with HIV to try LAI-ART? |

^a^The focus group questions for the topic, system/clinic/provider-related barriers/facilitators, does not pertain to consumer stakeholders.
